# Migratory Stress and living conditions abroad: Determinants of Cannabis use among migrants with psychiatric disorders

**DOI:** 10.1192/j.eurpsy.2026.10819

**Published:** 2026-07-31

**Authors:** F. Sahnoun, S. Omri, N. Smaoui, N. Charfi, I. Gassara, R. Feki, M. Maalej, M. Maalej Bouali, L. Zouari

**Affiliations:** Psychiatry C Department, Hedi Chaker University Hospital, Sfax, Tunisia

## Abstract

**Introduction:**

Precarious living conditions during migration represent a major stress factor that can promote substance use. Among migrants with psychiatric disorders, this relationship remains poorly explored.

**Objectives:**

This study aims to assess the association between living conditions abroad and cannabis use.

**Methods:**

This cross-sectional descriptive and analytical study was conducted among patients hospitalized in the Psychiatry “C” Department of Hédi Chaker University Hospital in Sfax. The study population included individuals who had previously lived abroad and whose psychiatric disorder began during their stay in a foreign country. Data collection took place between August and December 2024.

A structured questionnaire was used to collect sociodemographic data, as well as information related to migration and cannabis use.

**Results:**

A total of 39 male immigrants participated in the study. The mean age was 35.9 years. Among them, 28.2% were married and 20.1% had children.

Overall, 69.2% reported using cannabis during their stay abroad. The average duration of stay abroad was 82.6 months, and the mean age at migration was 22.5 years. In total, 69.2% had traveled illegally, 64.1% did not obtain legal status, and 46.2% were deported to their country of origin.

Financial difficulties were reported by 76.9% of participants, and 30.8% experienced homelessness during their stay. Only 30.8% had migrated with a family member.

Cannabis use was significantly associated with being single (p = 0.014), having no children (p = 0.043), irregular migration (p = 0.019), homelessness in the host country (p = 0.044), and migrating alone or with a friend (p = 0.014).

**Conclusions:**

Migratory stress and adverse living conditions as well as personal factors such as being single and having no children are key vulnerability factors in cannabis use among migrants with psychiatric disorders. These findings highlight the importance of an integrated care approach addressing both psychosocial context and migratory background.

**Disclosure of Interest:**

None Declared

